# Levels of Robotic Mesopancreas Dissection According to Malignancy and Vascular Anatomy: What Surgeons Need to Know

**DOI:** 10.1245/s10434-023-14686-8

**Published:** 2023-12-10

**Authors:** Benedetto Ielpo, Maria Vittoria d’Addetta, Alessandro Anselmo, Edoardo Rosso, Vito de Blasi, Patricia Sanchez-Velazquez, Gemma Vellalta, Mauro Podda, Fernando Burdio

**Affiliations:** 1 Hepato-Biliary and Pancreatic Surgery Unit, Department of Surgery, Hospital Parc Salut Mar, Pompeu Fabra University, Barcelona, Spain; 2 Hepato-Biliary Surgery, Borgoroma Hospital, Verona, Italy; 3 HPB and Transplant Surgery Unit, Department of Surgery, Policlinico Tor Vergata, Rome, Italy; 4 Unité des Maladies de l’Appareil Digestif et Endocrine, Centre Hospitalier de Luxembourg, Luxembourg City, Luxembourg; 5 Department of Surgical Science, University of Cagliari, Cagliari, Italy

**Keywords:** Pancreatoduodenectomy, Robotic, Mesopancreas

## Abstract

**Introduction:**

The robotic approach is attracting increasing interest among the surgical community, and more and more series describing robotic pancreatoduodenectomy have been reported. Thus, surgeons performing robotic pancreatoduodenectomy should be confident with this critical step’s potential scenarios.

**Materials and Methods:**

According to Yosuke et al., there are three different levels of mesopancreas dissection. We describe the main steps for a safe mesopancreas dissection by robotic approach.

**Results:**

This multimedia article provides, for the first time in literature, a comprehensive step-by-step overview of the mesopancreas dissection during robotic pancreatoduodenectomy (PD) and its three different levels according to tumor type.

**Conclusions:**

Through the tips and indications presented in this multimedia article, we aim to familiarize surgeons with the mesopancreas dissections levels according to type of malignancy and vascular anatomy.

**Supplementary Information:**

The online version contains supplementary material available at 10.1245/s10434-023-14686-8.

Given the high risk of hemorrhage secondary to the number of veins and arteries and their anatomical variants in this region, the mesopancreas dissection is a challenging and potentially dangerous phase of pancreatoduodenectomy (PD).^1^ Thus, pancreatic surgeons should be familiar with the different levels of its dissection according to the tumor and vascular variants.^2–4^ Because of this, different maneuvers designed to achieve an oncological and safe mesopancreas dissection exist.^5–7^

The robotic approach is attracting increasing interest among the surgical community, and more and more series describing robotic PD have been reported.^1,8^ Thus, surgeons performing robotic PD should be confident with this critical step’s potential scenarios. According to the pancreatic head malignancy, Yosuke et al. proposed three different levels of mesopancreas dissection for the open approach for the first time in 2015.^2^

This video article provides a comprehensive step-by-step overview of the mesopancreas dissection during robotic PD and its three different levels according to tumor type as described by Yosuke et al.^2^

## Anatomical Background

The mesopancreas is a firm and well-vascularized peripancreatic structure composed of fatty tissue with vascular structures, nerves, lymph nodes, and lymphatic vessels.^9–11^

As shown in the video, the mesopancreas is delimited laterally by the medial and posterior border of the uncinate process, medially by the right border of the superior mesenteric vein (SMV) and superior mesenteric artery (SMA); proximally by the origin of the celiac trunk and distally by the beginning of the mesenteric root, and finally, posteriorly by the left renal vein.

## Mesopancreas Dissection Main Steps

Before starting the mesopancreas dissection, we suggest dissecting the SMA first, as shown in the video, using a posterior approach. The artery’s origin can be easily identified in the axilla between the inferior vena cava and the left renal vein. Its dissection at this stage will make the mesopancreas dissection much safer, avoiding any damage. This step is crucial if a replaced right hepatic artery rises from the superior mesenteric artery, such as in the video.

As shown in the video, the first phase is the traction of the first jejunal loop, already dissected in its part proximally to the Treitz ligament, and then its transection using a mechanical stapler.

The second phase starts with separating the uncinate process from the mesentery of the first jejunal loop. It continues between the uncinate process and the terminal branch of the SMV. Here, a vessel-free space is present, where it is possible to work directly with scissors. The first jejunal branch of the SMV is dissected and sacrificed to facilitate the exposure of the SMA. Once identified, the uncinate process is dissected off the SMA in a caudal to cranial approach.

In the superficial dissection of the mesopancreas, the inferior pancreaticoduodenal vein (IPDV) is found first. In this case, for its dissection, LigaSure is used. If the IPDV is larger, a “hand-tight” ligature is used. The robotic platform offers an undoubted advantage whenever a ligature is needed.

The deep dissection of the mesopancreas is continued looking at the inferior pancreaticoduodenal arteries (IPDA) to be transected. The robotic VesselSealer and radiofrequency devices can be used to control bleeding at this stage.

## Mesopancreas Dissection Level 1

The first dissection level is used in benign and low-grade tumors, where a complete dissection of the mesopancreas is not always required.^2^

In this phase, it is essential to know the anatomical variations of the IPDV, which can arise directly from the first jejunal vein or the SMV.^2,3^

The SMV is dissected after the section of Henle’s gastrocolic trunk, the IPDV, and small pancreas tributaries. As shown in the video, the mesopancreas division is initiated caudally by retracting the SMV leftward. It ends anteriorly to the immediate right of the first jejunal artery, which is preserved.

Then the inferior pancreatic duodenal artery (IPDA) is exposed, ligated, and divided. The VesselSealer, radiofrequency instrument, and the “hook” cautery can be used for dissection.^3^

In this fashion, the SMV is isolated, leaving a posterior portion of mesopancreatic tissue, preserving the jejunal arteries and mesojejunum, and representing the limit of this type of mesopancreatic dissection.

## Mesopancreas Dissection Level 2

The second level is used in case of malignant tumors, where complete dissection of the mesopancreas is paramount to allow an oncological resection. In this case, it is vital to know the anatomical variations of arteries, such as the direct origin of the IPDA from the SMA or the first jejunal artery.^2^ The SMV is isolated after dissection and sacrifice of its tributaries, including the IPDV, the first jejunal vein, the colic vein, and Henle’s gastrocolic trunk. Then the IPDA and first jejunal artery are ligated and divided. In this type of dissection, the SMA is dissected, preserving the neurovascular bundle of the artery. Different types of energy devices can be used in this step, such as the Maryland bipolar dissector to identify the arterial and venous branches and the VesselSealer for a smooth dissection of the periarterial tissue. Thus, the mesopancreas and the mesojejunum are resected en bloc.

In our opinion, in case of intrapapillary mucinous neoplasm, this approach should be performed whenever a suspicious of degenerated lesion exists. However, it is not clear which level approach of mesopancreas dissection should be chosen.

## Mesopancreas Dissection Level 3

Type 3 is the most extended dissection, in which the mesopancreas is completely extirpated en bloc with the tissue surrounding the SMA. This is used in aggressive malignant tumors, where a certain grade of tissue infiltration can exist, such as borderline cases that underwent neoadjuvant treatment.^2^

This is described as the “anterior approach” to the SMA.^7^ The dissection is conducted up to the left side of the SMV. After that, the left side of the SMV is reached using a VesselLoop to control it. Then the IPDA and the first jejunal artery are ligated and divided, and the SMA is dissected just outside its adventitial plane. This is a technique described by Buchler’s group called “triangular operation” because it allows for a complete cleaning of the tissue between the SMV, the SMA, and the hepatic artery. This leads to the complete extirpation of the mesopancreas.^12^

This dissection is also mostly helpful in a venous resection, controlling the proximal and distal portions of the SMV and splenic vein with a BullDog clamp, allowing for a safe reconstruction, as shown in the video.

We use a three-dimensional (3D) reconstruction of preoperative imaging of our patients to improve surgical performance. A 3D reconstruction of preoperative patient imaging is of utmost importance in this approach as it can identify anomalies such as a replaced right hepatic artery in certain patients. We strongly suggest its use before any PD surgery.

## Conclusions

This multimedia manuscript provides a step-by-step comprehensive overview, tips, and indications to familiarize surgeons with all the possible robotic techniques for safe mesopancreas dissections levels. It is imperative to consider the specific indications for each strategy when performing a robotic mesopancreas dissection. This is because many factors are expected, including an aberrant artery, tumor location, and progression. Hence, surgeons attempting this approach should be well versed in the various strategies, and surgical anatomy should be recognized in each step.

### Supplementary Information

Below is the link to the electronic supplementary material.

Supplementary file1 (MP4 195813 kb)
